# *Citri Reticulatae Pericarpium* (Chenpi) Protects against Endothelial Dysfunction and Vascular Inflammation in Diabetic Rats

**DOI:** 10.3390/nu14245221

**Published:** 2022-12-07

**Authors:** Yuehan Wang, Xutao Zhang, Chunxiu Zhou, Haroon Khan, Manqin Fu, Wai San Cheang

**Affiliations:** 1 Institute of Chinese Medical Sciences, State Key Laboratory of Quality Research in Chinese Medicine, University of Macau, Macao SAR 999078, China; 2 Department of Pharmacy, Abdul Wali Khan University Mardan, Mardan 23200, Pakistan; 3 Sericultural & Agri-Food Research Institute, Guangdong Academy of Agricultural Sciences/Key Laboratory of Functional Foods, Ministry of Agriculture and Rural Affairs/Guangdong Key Laboratory of Agricultural Products Processing, Guangzhou 510610, China

**Keywords:** tangerine peel, diabetes mellitus, endothelial dysfunction, inflammation, AMPK

## Abstract

Dried tangerine peel (*Citri reticulatae Pericarpium*, CRP; Chenpi in Chinese) possesses medicine and food homology with hypolipidemic, anti-inflammatory and antioxidant activities. This study aimed to explore the protective effect of CRP extract on endothelial function and inflammation in type 2 diabetic rats and the related mechanisms. Type 2 diabetes mellitus was induced by high-fat diet (HFD)/streptozotocin (STZ) in male Sprague Dawley rats, and CRP extract was orally administered at 400 mg/kg/day for 4 weeks. Rat and mouse aortas were treated with high glucose and CRP extract ex vivo. The data showed that the ethanolic extract of CRP normalized blood pressure and the plasma lipid profile as well as the plasma levels of liver enzymes in diabetic rats. Impaired endothelium-dependent relaxations in aortas, carotid arteries and renal arteries were improved. CRP extract suppressed vascular inflammatory markers and induced AMPK activation in aortas of diabetic rats. Exposure to high glucose impaired vasodilation in aortas of rats and mice, and this impairment was prevented by co-incubation with CRP extract. In conclusion, our findings suggest that CRP extract protects endothelial function by inhibiting the vascular inflammatory state on activation of AMPK in diabetic rats.

## 1. Introduction

Cardiovascular disease is the leading cause of mortality and morbidity in patients with diabetes mellitus [1]. In addition, patients with chronic inflammatory diseases have higher chances of developing atherosclerosis and cardiovascular diseases, and diabetes mellitus as an inflammatory disease causes a two-fold increase in the risk of death from coronary heart disease, stroke and other vascular diseases [2]. Endothelial dysfunction and inflammation are commonly considered as interrelated and are crucial factors in the development of cardiovascular complications during diabetes progression [3]. The hyperglycemic, hypertensive, and hyperlipidemic conditions under diabetes contribute to vascular inflammation [4,5]. Inflammatory burden thereby leads to endothelial dysfunction, impairing endothelium-dependent dilatation [6].

High glucose levels in the blood circulation induce the expression of the pro-inflammatory enzymes inducible nitric oxide synthase (iNOS) and cyclooxygenase 2 (COX-2), which leads to endothelial cell apoptosis, disrupting endothelial function [7]. The adhesion molecules intercellular adhesion molecule 1 (ICAM-1) and vascular cell adhesion protein 1 (VCAM-1) are highly expressed in endothelial cells, accompanied by an increased density of inflammatory infiltrating cells during the vascular inflammation associated with the progression of cardiovascular diseases [8]. Adenosine monophosphate-activated protein kinase (AMPK) is a critical energy sensor and bioenergy regulator [9]. Moreover, extensive evidence supports that the activation of AMPK protects vascular function and suppresses vascular inflammation in diabetes through multiple pathways [10,11,12].

Dried tangerine peel (*Citrus reticulatae Pericarpium,* CRP; *Chenpi* in Chinese), is a traditional Chinese medicine with medicinal and food properties. It has the effects of regulating qi, invigorating the spleen, regulating dryness and dampness, and resolving phlegm [13]. Its main components are volatile oils such as d-limonene and flavonoid components such as hesperidin, naringenin, nobiletin, tangeretin, etc. In addition, polysaccharides, alkaloids and other components are also present [14]. Modern pharmacological studies have demonstrated that tangerine peel has various beneficial effects including hepatoprotective [15], anti-asthma [16], antioxidant [17,18], anti-inflammatory [19], and hypolipidemic activities [20]. In addition, flavonoids from tangerine peel were found to ameliorate metabolic and vascular dysfunction. Flavonoids including naringenin, naringin and nobiletin possess anti-atherogenic effects [21]. Nobiletin also exhibits vasodilatory effects in rat aortas [22]. To date, the possible protective effect of consuming dried tangerine peel in alleviating vascular inflammation and endothelial dysfunction in diabetes remains to be investigated.

Feeding with a high-fat diet (HFD) together with low doses of streptozotocin (STZ) is shown to induce type 2 diabetes in rats [23]. In this study, we aimed to investigate whether CRP extracts have a protective effect against endothelial dysfunction and vascular inflammation associated with diabetes in a rat model.

## 2. Materials and Methods

### 2.1. Preparation of CRP Extract

The tangerine peels of *Citrus reticulatae Pericarpium* (CRP) were collected in Xinhui, Guangdong Province, China, in 2016. The fresh peels were dried in a heat pump dryer (GHRH-20, Guangdong Agri-machinery Research Institute, Guangzhou, China) at 45 °C. Then the milled powder of tangerine peels was ultrasonically extracted 3 times with 80% EtOH (1:10 *w*/*v*) for 30 min each time. The filtrates were combined, and the solvent was recovered by rotary evaporation under reduced pressure, followed by lyophilisation with a Virtis Freeze Dryer (Te Virtis Company, New York, NY, USA) to obtain the final freeze-dried powder. Characterization of chemical components in CRP extract was determined by ultra-performance liquid chromatography-mass spectrometry (UPLC-MS) and the contents of hesperidin and hesperetin were quantified as reported in our previous study [19].

### 2.2. Animal Protocols

A total of 15 male Sprague Dawley (SD) rats at 8 weeks old were randomly divided into 3 groups, 5 rats for each group. All the rats were housed at a constant temperature (22–23 °C) with alternating 12-h light/12-h dark cycle with water ad libitum. The rats in the control group were fed a normal rodent diet; while rats in the diabetic (DM) and treatment (DM + CRP) groups were fed on a high-fat diet containing 45 kcal% fat for 12 weeks plus a single injection of low-dose streptozotocin (STZ, 35 mg/kg body weight, i.p.) at week 10 to induce type 2 diabetes. Then, rats in the treatment group were given CRP aqueous solution (400 mg/kg body weight) once daily for 4 weeks by oral gavage, whereas rats in DM and control groups were given distilled water. The body weights of the rats were measured biweekly. The weights of normal chow diet and high-fat diet consumed by each group of rats were also measured.

### 2.3. Oral Glucose Tolerance Test (OGTT)

The rats were loaded with glucose aqueous solution (2 g/kg body weight) by oral gavage after 12-h fasting. Afterwards, blood was taken from the tails of the rats to measure the blood glucose levels at 0, 15, 30, 60, 90 and 120 min by commercial glucometer (Jiangsu Yuyue Medical Equipment and Supply Co. Ltd., Zhenjiang, China). OGTT was performed at the end point of the experiment (after 4-week CRP administration).

### 2.4. Blood Pressure Measurement

Systolic (SBP) and diastolic (DBP) blood pressures were measured by the CODA noninvasive blood pressure system (a tail-cuff method, Kent Scientific Corporation, Torrington, CT, USA) in the three groups of conscious rats at the end of the experiment. The values of SBP and DBP were reported as the average of five successive measurements.

### 2.5. Determination of Plasma Lipid Profile

After the rats were sacrificed by CO_2_ inhalation, blood was drawn from the inferior vena cava and collected in heparin-coated microcentrifuge tubes. Plasma was obtained after centrifugation at 3000 rpm at 4 °C for 10 min and stored at −80 °C until further analysis. Plasma levels of total cholesterol (TC), triglyceride (TG), high-density lipoprotein cholesterol (HDL-C) and low-density lipoprotein cholesterol (LDL-C) were quantified using commercial kits according to the manufacturer’s instructions (Nanjing Jiancheng Bioengineering Institute, Nanjing, China, catalog numbers A111-1-1, A110-1-1, A112-1-1 and A113-1-1).

### 2.6. Determination of Liver Function Biomarkers in Plasma

The plasma levels of alanine aminotransferase (ALT) and aspartate aminotransferase (AST) as biomarkers for liver damage were measured using commercial kits according to the manufacturer’s instructions (Nanjing Jiancheng Bioengineering Institute, Nanjing, China, catalog numbers C009-2-1 and C010-2-1).

### 2.7. Ex vivo Culture of Rat and Mouse Aortas

Rat thoracic aortic segments were isolated and dissected free of adhering connective tissue in sterile phosphate buffered saline (PBS) and subsequently incubated in RPMI 1640 medium with additions of 10% fetal bovine serum (FBS) and 1% penicillin/streptomycin (Gibco, Grand Island, NY, USA), at 37 °C in a 5% CO_2_ environment. The aortic segments were supplemented with high glucose (44 mM) for 5 h to mimic hyperglycemic conditions in diabetes, while some were co-treated with 400 μg/mL CRP extract. The control group was cultured at the normal glucose level of the culture medium (11 mM) with addition of mannitol at the same volume as the osmotic control.

Thoracic aortic segments from C57BL/6J mice were also isolated in sterile PBS and subsequently incubated in Dulbecco’s Modified Eagle’s Medium (DMEM) with 10% FBS and 1% penicillin/streptomycin at 37 °C in a 5% CO_2_ environment. Some of the aortas were additionally supplemented with 30 mM glucose to simulate a high-glucose environment, with or without the co-treatment of 400 μg/mL CRP extract. The control group was cultured at the normal glucose level of the culture medium (5.55 mM) plus the same volume of mannitol as the osmotic control.

### 2.8. Isometric Force Measurement in Wire Myograph

Arterial segments including aortas, carotid arteries and renal arteries from the SD rats as well as aortas from C57BL/6J mice were cut to ~ 2 mm in length and were suspended in a Multi Myograph System (Danish Myo Technology, Denmark) for a functional study to detect changes in isometric tension. The blood vessel segments were stretched to optimal baseline tension and were equilibrated for 60 min, followed by induction of vasoconstriction using 60 mM KCl. Endothelium-dependent relaxations (EDRs) were studied in response to cumulative addition of acetylcholine (ACh, 3 nM-10 μM, Sigma-Aldrich, St. Louis, MO, USA) upon precontraction of phenylephrine (Phe, Sigma-Aldrich) in endothelium-intact rings. Then sodium nitroprusside (SNP, 1 Nm–10 μM, Sigma-Aldrich)-induced endothelium-independent relaxations were measured in Phe-contracted rings. Each experiment was performed on rings prepared from different experimental animals.

The baseline and Phe concentrations were at 10 mN and 1 µM for rat aortas, 5 mN and 3 μM for rat carotid arteries, 2.5 mN and 1 μM for rat renal arteries, and 3 mN and 3 µM for mouse aortas.

### 2.9. Western Blotting

Aortas from rats after chronic treatment were isolated and snap frozen in liquid nitrogen. They were subsequently homogenized in ice-cold RIPA lysis buffer (Beyotime Biotechnology, Shanghai, China) plus cOmplete protease inhibitor cocktail (Roche, Basel, Switzerland) and PhosSTOP phosphatase inhibitors (Roche, Basel, Switzerland). The lysates were incubated on ice for 30 min and then centrifuged at 15,000 rpm for 30 min. The supernatant was collected and the protein concentration was measured using the bicinchoninic acid (BCA) assay (Beyotime Biotechnology, Shanghai, China). The protein samples (15 μg) were electrophoresed through 10% sodium dodecyl sulfate polyacrylamide gel electrophoresis (SDS-PAGE) and transferred to PVDF membranes (Millipore, Billerica, MA, USA) using wet transfer. Non-specific binding sites were blocked with 1% BSA in 0.05% Tween-20 PBS, and then the membrane was probed overnight at 4 °C with a primary antibody against the target protein including, iNOS, COX-2, VCAM-1, ICAM-1, phosphor (p)-AMPKα at Thr172 and AMPKα (Cell Signaling Tech, Danvers, MA, USA). The blots were incubated with the corresponding HRP-coupled secondary antibody (Beyotime Biotechnology, Shanghai, China) for 1 h at room temperature. GAPDH was selected as the housekeeping protein and used to check the equal loading of each sample. Protein bands were finally developed with the American ECLTM Advanced Western Blotting Detection Kit (GE Healthcare Life Sciences, Uppsala, Sweden), and images were captured using the ChemiDoc^TM^ MP Imaging System (Bio-Rad, Hercules, CA, USA). The intensities of signals were analyzed by ImageJ software (National Institute of Health, Bethesda, MA, USA).

### 2.10. Statistical Analysis

All data were shown as mean ± standard error of mean (SEM) of n-independent experiments. Comparisons among groups were analyzed using one-way ANOVA followed by Bonferroni post hoc tests and Student’s *t*-test in the GraphPad Prism software (GraphPad Software, San Diego, CA, USA). *p* < 0.05 was considered as a statistically significant difference.

## 3. Results

### 3.1. CRP Extract Mitigates High Blood Pressure in Diabetic Rats

**Figure 1:**
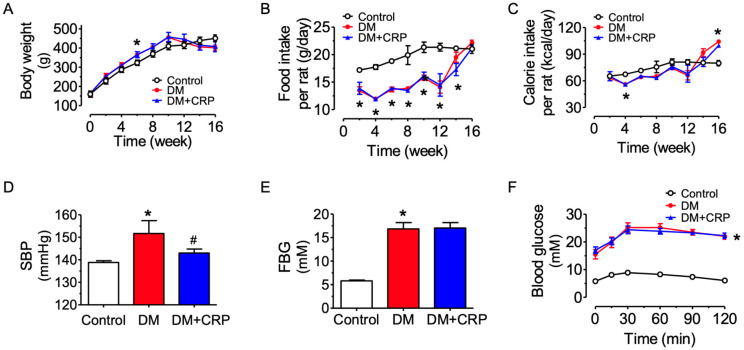
Effects of tangerine peel (*Citrus reticulatae Pericarpium*, CRP) extract administration on body weight, blood pressure and blood glucose level of diabetic rats. (**A**) Body weight changes. (**B**,**C**) Food and energy intake. (**D**) Systolic blood pressure (SBP) measured by tail-cuff method. (**E**) Fasting blood glucose (FBG) measured after 12 h of fasting. (**F**) Oral glucose tolerance test (OGTT) after 12 h of fasting. Data are mean ± SEM from five rats for each group. * *p* < 0.05 DM vs. Control; # *p* < 0.05 DM + CRP vs. DM.

Male Sprague Dawley rats were randomly assigned into three groups which had similar body weight at the start of the experiment, and rats feeding on a high-fat diet (HFD) had a moderately higher increase in body weight than the control group feeding on normal chow (Figure 1A). The food intake in terms of weight for consumption of HFD was less than that of normal chow by the control rats (Figure 1B); nevertheless, the calorie intake was similar among the groups (Figure 1C). After streptozotocin (STZ) injection at week 10, the body weight in the diabetic rats was reduced, whilst the food/calorie intake was increased. The CRP treatment (400 mg/kg/day, 4 weeks) had no effect on body weight and food/calorie intake in the diabetic rats. The blood glucose levels of the model rats were significantly higher at each time point than those of the control rats (Figure 1E,F). The systolic blood pressure (SBP) in HFD/STZ-induced diabetic rats was significantly increased, and the hypertensive condition was significantly mitigated by CRP treatment (Figure 1D). However, the blood glucose level was not altered by CRP treatment, as indicated in the results of the fasting blood glucose (FBG) determination and oral glucose tolerance test (Figure 1E,F). The elevated FBG and glucose intolerance supported that the type 2 diabetes model was successfully induced in rats.

### 3.2. CRP Extract Ameliorates Lipid Metabolism Dysregulation and Liver Function Injury in Diabetic Rats

**Figure 2:**
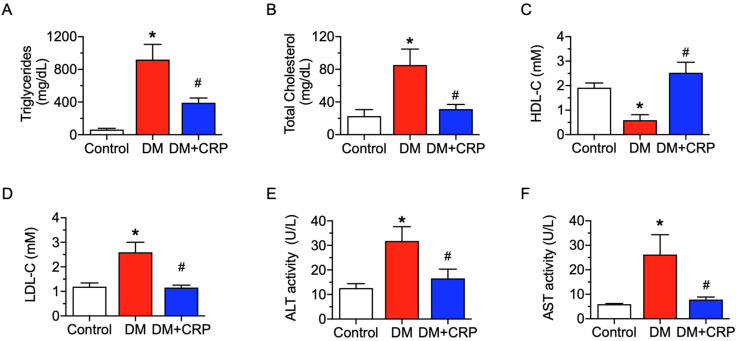
Effect of CRP extract on plasma lipid profile and liver function biomarkers in rats. Levels of (**A**) triglycerides, (**B**) total cholesterol, (**C**) high-density lipoprotein cholesterol (HDL-C), (**D**) low-density lipoprotein cholesterol (LDL-C), and (**E**) alanine aminotransferase (ALT) and (**F**) aspartate transaminase (AST) activities detected in plasma. Data are mean ± SEM from five rats for each group. * *p* < 0.05 DM vs. Control; # *p* < 0.05 DM + CRP vs. DM.

At 16 weeks, the diabetic rats exhibited significant abnormalities in lipid metabolism, including significant increases in plasma levels of triglycerides, total cholesterol, low-density lipoprotein (LDL-C), and significant decreases in high-density lipoprotein (HDL-C), and such changes were remarkably reversed by four-week CRP administration (Figure 2A–D). Similarly, plasma AST and ALT activities were diminished in the CRP-treated group (Figure 2E,F), implying that the liver function damage of diabetic rats was significantly improved after chronic CRP treatment.

### 3.3. CRP Extract Alleviates Endothelial Dysfunction Associated with Diabetes

**Figure 3:**
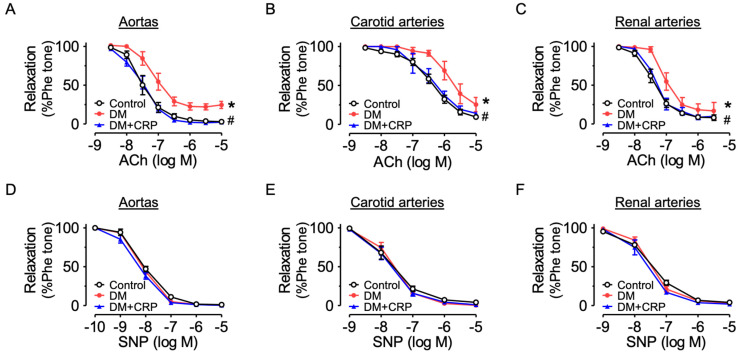
Vascular protective effects of CRP extract on diabetic rats. (**A**–**C**) Effects of oral administration of CRP (400 mg/kg/day for 4 weeks) on acetylcholine (ACh)-induced endothelium-dependent relaxations in the aortas, carotid arteries and renal arteries of diabetic rats. (**D**–**F**) Sodium nitroprusside (SNP)-induced endothelium-independent relaxations in the aortas, carotid arteries and renal arteries of diabetic rats. Data are mean ± SEM from five rats for each group. * *p* < 0.05 DM vs. Control; # *p* < 0.05 DM + CRP vs. DM.

Aortas, carotid arteries and renal arteries of the control and diabetic rats were dissected for vascular functional assays. Compared with the arteries from the control group, the acetylcholine (ACh)-induced endothelium-dependent relaxations (EDRs) were markedly reduced in diabetic rats (Figure 3A–C). Treatment with CRP extract at 400 mg/kg/day for 4 weeks significantly reversed the impairments in aortas, carotid arteries and renal arteries. On the other hand, diabetes or CRP treatment did not affect the SNP-induced endothelium-independent relaxations (Figure 3D–F), indicating the vascular smooth muscle function responding to NO was unaltered.

### 3.4. CRP Extract Inhibited Inflammation in the Aortas of Diabetic Rats

**Figure 4:**
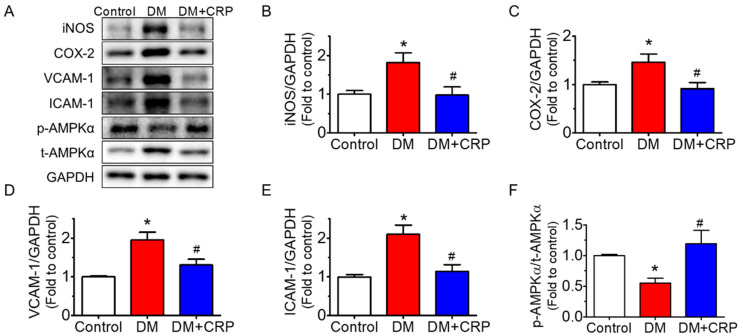
Effect of CRP extract on the protein expressions of inflammation markers in the rat aortas. (**A**) Representative blots and (**B**–**F**) summarized data showing the effect of CRP extract treatment (400 mg/kg body weight, 4 weeks) on aortic inflammation in diabetic rats, including iNOS (130 kDa), COX-2 (74 kDa), VCAM-1 (110 kDa), and ICAM-1 (90 kDa) compared with GAPDH (36 kDa) as well as the phosphorylation of AMPKα at Thr172 (p-AMPKα; 62 kDa) compared with its total protein (t-AMPKα). Data are mean ± SEM from five rats for each group. * *p* < 0.05 DM vs. Control; # *p* < 0.05 DM + CRP vs. DM.

The protein expressions of common inflammatory markers such as iNOS, COX-2, VCAM-1 and ICAM-1 were significantly elevated (Figure 4A–E), whereas the phosphorylated AMPKα at Thr172 was suppressed in the aortas of the diabetic rats (Figure 4A,F). These changes in protein expression were reversed after 4 weeks of CRP extract treatment. The GAPDH levels indicated equal loading of each sample in the Western blot analysis.

### 3.5. CRP Extract Protects against Endothelial dysfunction in Hyperglycemic conditions

**Figure 5:**
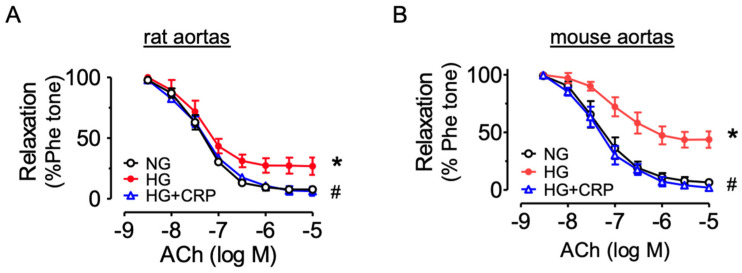
Vasoprotective effect of CRP in aortas from SD rats and C57BL/6J mice ex vivo. Effect of CRP extract (400 µg/mL) on acetylcholine (ACh)-induced endothelium-dependent relaxations of aortas (**A**) from rats exposed to high glucose (HG, 44.4 mM, 5 h) and (**B**) from mice (30 mM, 48 h). Data are mean ± SEM of 4 experiments. * *p* < 0.05 HG vs. NG; # p < 0.05 HG + CRP vs. HG.

Furthermore, we confirmed the protective effect of CRP extract on both rat and mouse aortas ex vivo. The rat aortas were incubated with high glucose (44.4 mM, 5 h) to mimic the hyperglycemic environment in diabetes. Ex vivo high glucose exposure impaired the ACh-induced EDRs compared with the normal glucose control (11.1 mM, 5 h with mannitol added as a osmotic control), and the impairment was reversed by CRP co-treatment at 400 µg/mL (Figure 5A). Similarly, CRP extract prevented high glucose (30 mM, 48 h)-induced EDR impairment in mouse aortas (Figure 5B).

## 4. Discussion

The present results suggest that the CRP extract has a significant protective effect against vascular inflammation and endothelial dysfunction associated with diabetes on AMPK activation. We observe that HFD/STZ-induced diabetic rats have high blood pressure, increased levels of plasma lipids and liver enzymes, upregulated inflammatory markers in aortas, and impaired vasodilation in thoracic aortas, carotid arteries and renal arteries, which are reversed by chronic administration of CRP extract in vivo.

Previous studies have demonstrated that different types of citrus peel extracts exert beneficial effects on metabolic disorders, including lowered body weight, improved hyperglycemia, reduced dyslipidemia and reduced liver function abnormalities in obese and diabetic mice [24,25,26,27]. In consistence with previous studies, the current study shows that chronic consumption of CRP extract decreases plasma levels of total cholesterol, triglycerides, LDL, AST, ALT and increases plasma HDL level in diabetic rats. However, our data illustrate no significant changes in body weight and glucose tolerance with oral treatment of CRP extract, which contradicts previous studies. These contradictions may be attributed to the difference in treatment dosage, duration, and animal models used.

Fresh citrus peel has been shown to protect against atherosclerosis, reducing the fatty plaque in coronary arteries and aortas and enhancing the antioxidant capacity in rabbit plasma [28]. The polymethoxy-flavonoid nobiletin present in citrus peel significantly improves hemodynamic parameters, oxidative stress, collagen levels, vascular reactivity, cardiac hypertrophy index and myocardial fibrosis in STZ-induced diabetic rats [29]. In addition, this compound exerts endothelium-independent vasodilatory effects to attenuate phenylephrine-induced constriction in rat aortas by increasing cyclic guanosine monophosphate (cGMP) levels through guanylate cyclase activation [22]. These previous findings suggest that tangerine peel and/or its ingredients may affect vascular function. Our present study is probably the first to demonstrate that CRP has a good vascular protective effect in mitigating high blood pressure and enhancing endothelium-dependent vasodilation in conduit arteries such as the aortas, carotid arteries and renal arteries in diabetic rats in vivo. Meanwhile, the endothelial-independent relaxations induced by cumulative addition of NO donor SNP in the arteries were unaltered, implying a normal vascular smooth muscle function [30]. The same improvement in EDRs was observed in isolated aortas from both rats and mice upon exposure to high glucose ex vivo. Notably, the beneficial effect of restoring lipid profiles in diabetic rats might partially mediate the protective properties of CRP extract in vasculature. However, the ex vivo experimental evidence suggests that the contribution of lipid modulation should be minimal and that CRP extract possesses direct vasoprotective activity as ambient lipid levels are constant in the culture medium on isolated aortas.

Inflammatory processes actively participate in the development of vascular dysfunction associated with chronic metabolic diseases. Both obesity and diabetes are inflammatory conditions. Surplus concentrations of nutrients, such as glucose and free fatty acids (FFAs), are observed in obesity and type 2 diabetes [31]; in parallel, inflammation is found in various tissues including fat, liver, muscle, islets, and blood vessels [32,33,34,35,36]. Vascular risk factors including hyperglycemia, advanced glycation end products (AGEs), oxidized lipids found in diabetes and obesity can elevate the expressions of VCAM-1 and ICAM-1 and cause inflammation, and inflammation aggravates the progression of cardiovascular disease [37]. We have recently reported that CRP extract has anti-inflammatory effects in macrophages [19]. In addition, the main flavonoid components of CRP, consisting of hesperidin, nobiletin, and tangeretin, inhibit neuroinflammation [38]. Thus, it is reasonable to postulate that CRP might improve endothelial function through anti-inflammatory effects.

Our findings show that CRP restrained vascular inflammation, downregulating the expressions of iNOS, COX-2, ICAM-1, and VCAM-1 and activating AMPK in the aortas of HFD/STZ-induced diabetic rats. ICAM-1 and VCAM-1 are critical for macrophage activation and recruitment and strongly linked to inflammation. AMPK is a phylogenetically conserved serine/threonine protein kinase. AMPK regulates not only energy homeostasis but also vascular homeostasis. AMPK activation is widely considered to elicit anti-inflammatory effects and protect endothelial function in the vascular system [39]. AMPK can be stimulated by various natural and synthetic compounds, and AMPK modulates multiple targets. AMPK activation by adenine suppresses the inflammatory response in endothelial cells, where COX-2, ICAM-1, and VCAM-1 expressions are inhibited [40]. Activation of AMPK is reported to inhibit inflammation through phosphorylation of p300 and inactivation of p300 histone acetyltransferase, and thereby the nuclear factor kappa B (NF-κB) pathway [41]. Methotrexate alleviates inflammation in an AMPK-CREB-dependent manner [42]. Additionally, AMPK is suggested to trigger the biogenesis and maturation of microRNAs (miRNAs), thereby modulating vascular homeostasis [43,44]. Our data indicate that CRP extract ameliorates vascular inflammation and endothelial dysfunction in diabetes, possibly mediated by AMPK activation; nevertheless, the comprehensive mechanism underlying AMPK activation and the involvement of other proteins and signaling pathways need to be further explored in future study. Natural flavones are suggested to be protective against inflammation associated with diabetes [45]. CRP contains flavones such as hesperidin and other ingredients. Investigation and identification of the active ingredients present in CRP extract contributing to its vasoprotective effect should be performed in the future.

## 5. Conclusions

In conclusion, chronic administration of tangerine peel extract confers protection against vascular inflammation and endothelial dysfunction in arteries from diabetic rats, possibly via AMPK activation. CRP treatment also normalizes blood pressure and plasma lipid levels. These novel findings strengthen the prospects for the potential use of tangerine peel in functional foods or health supplements for patients with metabolic disorders and vascular complications.

## Data Availability

The data presented in this study are available on request from the corresponding author.
